# Impact of weight change since a young age on cardiovascular mortality risk: a pooled analysis of Japanese epidemiological evidence

**DOI:** 10.1265/ehpm.22-00002

**Published:** 2022-03-04

**Authors:** Ahmed Arafa, Rena Kashima, Yoshihiro Kokubo

**Affiliations:** 1 Department of Preventive Cardiology, National Cerebral and Cardiovascular Center, Suita, Japan; 2 Public Health, Department of Social Medicine, Graduate School of Medicine, Osaka University, Suita, Japan; 3 Department of Public Health, Faculty of Medicine, Beni-Suef University, Beni-Suef, Egypt; 4 Public Health Division, Ibaraki Public Health Center, Osaka Prefectural Government, Ibaraki, Japan

**Keywords:** Weight change, Pooled analysis, Cohort studies, Japan

Dear editor,

The relationship between weight change since a young age and the risk of cardiovascular disease (CVD) mortality among Japanese is not well-established. Herein, we conducted a quick literature search and review of studies investigating this association before combing the results of eligible studies in a pooled analysis.

First, we searched PubMed for potential studies published in English before the 10^th^ of December 2021 using the following terms: (weight change) AND (cardiovascular mortality) AND (Japan). Then, we conducted a manual search of the reference lists of retrieved articles to obtain additional studies. Our eligibility criteria included: 1) the exposure was weight change since a young age, 2) the outcome was CVD mortality, 3) the study had a prospective cohort design, and 4) the study investigated Japanese populations. We did not set limitations regarding publication year.

Eventually, four studies were eligible. The included studies used data from Japan Public Health Center-based prospective Study (JPHC) [1], the Ohsaki Study [2], Japan Collaborative Cohort Study (JACC) [3], and the Suita Study [4]. All studies were population-based with prospective cohort designs. The age ranges of participants were as follows: JPHC: 40–69 years, Ohsaki: 40–79 years, JACC: 40–79 years, and Suita: 30–79 years. Weight at age 20 was assessed using a question in the baseline questionnaires of the four studies asking participants to recall their weight at age 20. The definitions of maximum weight gain, maximum weight loss, and stable weight differed across the four studies; JPHC: ≥5, ≤−5, and ±4.9 kg, Ohsaki: ≥10, ≤−10, and ±4.9 kg, JACC: ≥12.5, ≤−12.5, and ±2.4 kg, and Suita >10, <−10, and ±4.9 kg, respectively. Mortality surveillance was conducted by systematically reviewing death certificates and CVD mortalities were assigned by ICD codes. The four studies had lengthy median follow-up periods: JPHC: 12.9 years, Ohsaki: 13.3 years, JACC: 19.1 years, and Suita: 19.9 years.

Then, we extracted the hazard ratios (HRs) with 95% confidence intervals (CIs) of CVD mortality for maximum weight gain and loss categories compared to stable weight categories in the most adjusted models. Later, we calculated the pooled HR (95% CI) of the four studies for weight gain and weight loss using the random-effects model, assuming that the underlying effects were different across studies [5]. Besides, we performed the *I*^2^ statistic to evaluate heterogeneity across studies and the test for funnel plot asymmetry to detect publication bias [6, 7]. The risk of bias was assessed by the first- and last-place authors using the modified Newcastle–Ottawa Scale based on studies’ selection, comparability, and outcome [8].

In the pooled analysis, maximum weight loss was associated with increased CVD mortality risk: 1.44 (1.31, 1.58). Maximum weight gain, on the other hand, was not associated with CVD mortality risk: 1.01 (0.83, 1.23). No signs of heterogeneity across studies were detected in the weight loss pooled analysis (*I*^2^ = 0.0%), but heterogeneity could be detected in the weight gain pooled analysis (*I*^2^ = 78.8%) (Fig. 1). No publication bias was identified in weight loss (z = 0.28 and p = 0.78) and weight gain (z = −0.84 and p = 0.40) pooled analyses. None of the included studies carried any significant risk of bias. Of note, JPHC, Ohsaki, and Suita stratified their results by age and concluded no significant impact of age on the association between weight change and CVD mortality.

**Fig. 1:**
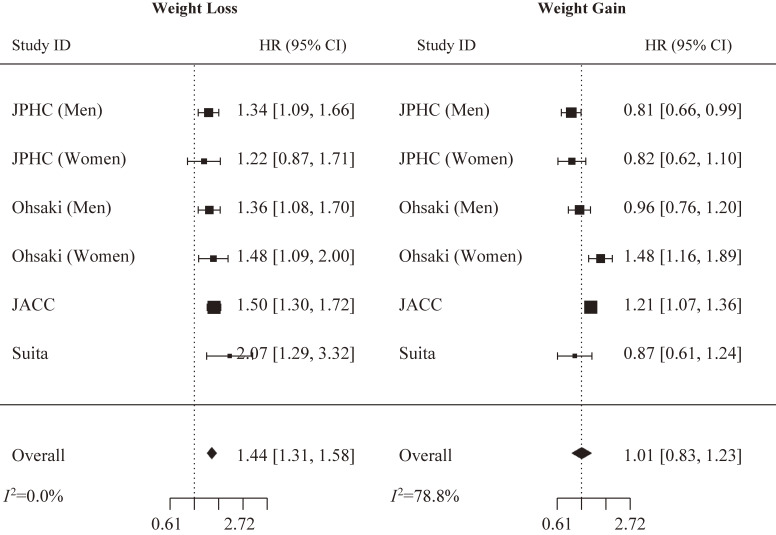
Weight change since age 20 and cardiovascular mortality risk

Our study concluded that excess weight loss since age 20 could be associated with the increased risk of CVD mortality among Japanese. Worsening chronic diseases, pre-existing medical conditions, sarcopenia, and loss of the beneficial peripheral subcutaneous fat were suggested as explanations for this association [1–4].

Still, some limitations should be considered. First, the long-term recall of weight might have carried a potential risk of recall bias in the four studies, yet strong correlations between recalled and measured weights were detected in a previous Japanese study [9], suggesting that recall bias did not materially affect the results. Second, the four studies did not clarify whether weight change was intentional or not. A previous meta-analysis suggested that unintentional, but not intentional, weight loss was associated with a higher risk of mortality [10]. Third, residual confounding cannot be excluded because of the observational nature of the included studies.

## Abbreviations

CI: Confidence Interval
CVD: Cardiovascular Disease
HR: Hazard Ratio
JACC: Japan Collaborative Cohort study
JPHC: Japan Public Health Center-based prospective study
